# Survival after radiofrequency ablation and/or chemotherapy for lung cancer and pulmonary metastases: a systematic review and meta-analysis

**DOI:** 10.3389/fimmu.2023.1240149

**Published:** 2023-10-06

**Authors:** Ziyi Yang, Xia Lyu, Huilin Yang, Bingbing Wang, Dan Xu, Lingyi Huo, Runzi Zhang, Yingjun Huang, Benshu Diao

**Affiliations:** Chengdu Seventh People’s Hospital & Chengdu Tumor Hospital, Chengdu, Sichuan, China

**Keywords:** lung cancer, pulmonary metastases, radiofrequency ablation, chemotherapy, systematic review, meta-analysis, RFA

## Abstract

**Background:**

Radiofrequency ablation (RFA) and chemotherapy are used to treat lung cancer or pulmonary metastases, but no direct comparison of overall survival (OS) has been published. The present study aimed to assess the OS of RFA and/or chemotherapy in patients with lung cancer or pulmonary metastases who were not candidates for surgical resection.

**Methods:**

To identify relevant studies, the following databases were electronically searched from their inception to 31 March 2023: PubMed, Embase, Web of Science, Cochrane Library, Scopus, Ovid, ScienceDirect, SinoMed, China National Knowledge Infrastructure Database, Chongqing VIP Chinese Science and Technology Periodical Database, Wanfang Database, LILACS, ClinicalTrials.gov, and Chictr.org. Manual retrieval was also conducted. We used published hazard ratios (HRs) if available or estimates from other survival data.

**Results:**

A total of 1,387 participants from 14 trials were included in the final analysis. Patients treated with RFA combined with chemotherapy significantly improved OS compared with those treated with chemotherapy alone [HR 0.50, 95% confidence interval (CI) 0.41–0.61; p < 0.00001], with an absolute difference at 12 months of 29.6% (95% CI 23.7–35.5), at 24 months of 19.2% (95% CI 10.1–28.2), and at 36 months of 22.9% (95% CI 12.0–33.7). No statistically significant difference was observed in the subgroups of case type, cancer type, chemotherapy drugs, and tumor size. The HR for OS with RFA plus chemotherapy vs. RFA alone was 0.53 (95% CI 0.41–0.70; p < 0.00001), corresponding to a 27.1% (95% CI 18.3–35.8), 31.0% (95% CI 19.9–41.9), and 24.9% (95% CI 15.0–34.7) absolute difference in survival at 12 months, 24 months, and 36 months, respectively. Subgroup analysis by geographic region and TNM stage showed that RFA combined with chemotherapy still significantly improved OS compared to RFA. The HR of RFA vs. chemotherapy was 0.98 (95% CI 0.60–1.60; p = 0.94), with an absolute difference at 12 months of 1.4% (95% CI -19.2 to 22.1), at 24 months of 7.8% (95% CI -11.3 to 26.8), and at 36 months of 0.3% (95% CI -13.2 to 13.8). The overall indirect comparison of OS for RFA vs. chemotherapy was 0.95 (95% CI 0.72–1.26; p = 0.74). Data on progression-free survival were not sufficiently reported.

**Conclusion:**

RFA combined with chemotherapy might be a better treatment option for patients with lung cancer or pulmonary metastases than chemotherapy alone or RFA alone. The comparison between RFA and/or chemotherapy remains to be specifically tested.

**Systematic review registration:**

https://www.crd.york.ac.uk/PROSPERO/display_record.php?RecordID=335032, identifier CRD42022335032.

## Introduction

In 2020, the estimated number of new cases of lung cancer diagnosed worldwide was 2,206,771 (11.4%) and lung cancer-related deaths were 1,796,144 (18.0%) (1). Lung cancer is the second most commonly diagnosed cancer and the top cause of cancer deaths globally (1, 2). Because the prophase of clinical symptoms is not obvious, over 70% of patients already have advanced disease at the time of presentation (3–5). The survival of lung cancer in advanced stages is still very poor, with an overall 5-year survival rate of 9.5%–18% (5, 6). Above 54%, patients with cancer may develop pulmonary metastases, for many tumors involve the lung for distant spread (5). In colorectal cancer, patients who present with lung metastasis have a 5-year survival rate of less than 10% compared with 91% of those without metastasis (7).

Surgical resection is preferred in the treatment of lung tumors and pulmonary metastases. However, a significant number of patients who are not candidates for surgical resection receive multidisciplinary synthetic treatment (8). Chemotherapy, as the main adjuvant method in the treatment of cancer, has been widely applied in advanced lung cancer treatment. Platinum-based chemotherapy is an essential part of the treatment of locally advanced lung cancer. Their effects on the survival of patients are still far from satisfactory because the median overall survival (OS) was only 9 months and the over 1-year survival rate was 30% (9). Radiofrequency ablation (RFA), a minimally invasive technique, has been gradually introduced for pulmonary tumor treatment in recent years. In a large prospective trial of RFA for lung cancer (10), there was no difference in response between primary and metastasized lung tumors. The OS of 1 year is 70% in patients with non-small cell lung cancer (NSCLC) and 89% at 1 year in patients with colorectal metastases. Combining systemic therapy with local therapy is always the focus of clinical inquiry. The combination of RFA with chemotherapy improves survival, offers higher treatment efficacy, and delays disease progression (11–13).

Three reviews and meta-analyses have been published on this subject (14–16). They focused on lung tumors in nonsurgical patients, short-term clinical effects, survival rate, local tumor progression, quality of life, recrudescence, and drug toxicity (14–16). However, all of the trials that were included in these meta-analyses were conducted before 2014. Furthermore, they did not evaluate survival by hazard ratio (HR). The limited scope of previous reviews and the recent publication of a number of studies assessing OS for patients treated with RFA and/or chemotherapy require a new comprehensive meta-analysis.

Our objective was to conduct a systematic review and meta-analysis to compare the survival of RFA and/or chemotherapy on lung cancer and pulmonary metastases in patients who are nonsurgical candidates and try to provide evidence in support of clinical work in choosing appropriate treatment options.

## Methods

We conducted and reported this systematic review and meta-analysis according to the Preferred Reporting Items for Systematic reviews and Meta-Analyses (PRISMA) guidelines (17). The protocol was registered with PROSPERO (registration number: CRD42022335032) at the start of our investigation.

### Research question

We aimed to evaluate the survival of RFA, chemotherapy, and RFA plus chemotherapy for patients with lung cancer and pulmonary metastases.

### Types of studies

We included randomized controlled trials (RCTs), cohort studies, and case-control studies. Case series lacking comparator groups or follow-up less than 12 months were excluded. Published and unpublished studies, full articles, and abstracts satisfying the criteria listed below were included without any language restriction. For publications and unpublished works not subject to peer review (such as theses or reports), we would contract the authors to get the key data. We also hand-searched the reference lists of the included studies and topical reviews for potentially relevant articles.

### Type of participant

We reviewed studies reporting on patients with lung cancer and/or pulmonary metastases who were not eligible for surgical resection and were receiving the treatment of RFA, chemotherapy, and RFA plus chemotherapy.

### Type of intervention

The arms of the studies were only chemotherapy, RFA, and RFA plus chemotherapy. Other adjunctive therapies (e.g., microwave ablation and radiotherapy) and targeted treatments were excluded.

### Type of outcome measure

The primary outcome was OS, defined as the time from randomization to death from any cause. The secondary outcome was progression-free survival (PFS), defined as the time from randomization to disease progression or death.

### Search strategy

We systematically searched the following databases from their date of inception to 31 March 2023: PubMed, Embase, Web of Science, Cochrane Library, Scopus, Ovid, ScienceDirect, SinoMed, China National Knowledge Infrastructure Database (CNKI), Chongqing VIP Chinese Science and Technology Periodical Database (VIP), Wanfang Database, LILACS, ClinicalTrials.gov, and Chictr.org. There were no language restrictions, and they carried out translations if necessary. The search strategy included four core components: 1) lung cancer; 2) pulmonary metastases; 3) chemotherapy; and 4) RFA. The retrieval model was ((a) OR (b)) AND (c) AND (d). Medical Subject Headings (MeSH) terms, free-text terms, keywords, and subject words were identified for each of the above core components. MeSH terms were retrieved from PubMed. The search was based on PubMed and then adapted for other English databases. For other databases, the subject-word retrieval method was used. The searching strategy is presented in **Supplementary Table S1**. References from previous reviews and key articles retrieved were also reviewed and cross-referenced for relevant studies.

### Selection of studies

We downloaded all titles and abstracts obtained by electronic searches to a reference management database (Microsoft Excel) and removed duplicate articles. The remaining titles and abstracts were independently reviewed by two authors. They excluded studies that clearly did not meet the inclusion criteria. We obtained full-text articles of the remaining articles, and two independent reviewers determined the eligibility of the retrieved papers. We resolved disagreements by consensus or by consulting the senior author if necessary. We documented reasons for exclusion during this process.

### Data extraction and management

Two review authors independently extracted the data using the extraction template, which include study characteristics (authors, journal, year of publication, location, and funding), study questions (participants, comparison, aims, design, follow-up time, type of study, and size), results (outcomes, key findings), and conclusions. When a consensus on the data extraction cannot be obtained through consultations, the senior author will make a decision.

### Assessment of risk of bias and reporting of study quality

Two reviewers independently evaluated the methodological quality of the included studies. For RCTs, we assessed the risk of bias and created applicability concerns graph using the Cochrane Risk of Bias Tool, which is structured into seven domains: random sequence generation, allocation concealment, blinding of participants and personnel, blinding of outcome assessment, incomplete outcome data, selective outcome reporting, and other sources of bias. The outcomes include low risk of bias, unclear risk of bias, and high risk of bias (18, 19).

For cohort and case-control studies, the Newcastle–Ottawa Scale (NOS) was used to assess the risk of bias (19). It assigns up to a maximum of nine points for the least risk of bias in three domains: 1) selection of the study groups (4 points); 2) comparability of groups (2 points); and 3) ascertainment of exposure and outcomes (3 points) (20). The maximum score of each study was 9. Studies with scores of 7 were considered to have a low risk of bias, scores of 4–6 were considered to have a moderate risk of bias, and scores <4 were considered to have a high risk of bias. We assessed that follow-up was adequate if the follow-up was in excess of 12 months. Any disputes will be settled via consensus or with the involvement of the senior author.

### Measures of treatment effect

We used the HR for the comparison in each trial to assess the treatment effects.

### Management of missing data

If there are missing data for the primary results, we will contact the corresponding authors to request the missing data. If the missing data cannot be obtained, the analysis will rely on the available data. HRs and 95% confidence intervals (CIs) not reported or supported were calculated by the survival curves (21).

### Statistical synthesis

The meta-analysis was performed using Review Manager (RevMan V.5.4.1 for Windows; the Nordic Cochrane Centre, The Cochrane Collaboration, 2020) and R version 4.2.1. The chi-square heterogeneity test and I^2^ statistic were used to investigate the overall heterogeneity between trials. p < 0.10 or I^2^ > 50% indicated significant heterogeneity. If considerable heterogeneity was observed, a random-effects model was used to analyze the pooled effect estimate; otherwise, the fixed-effects model was used. If more than 10 trials were included, funnel plots and the Egger test were used to assess publication biases. To estimate the 12-month, 24-month, and 36-month absolute differences, survival rates were computed on all patients and the HR at the corresponding time period was used to compute survival in each group (22). We used indirect comparison to obtain estimates of the benefit of RFA compared with chemotherapy.

### Subgroup analysis

When significant heterogeneity was found, subgroup and sensitivity analyses were performed to explore possible reasons for the heterogeneity. However, given that the main purpose of subgroup analyses was to assess differences between subgroups rather than to explore reasons for heterogeneity, we performed subgroup analyses regardless of the presence or absence of statistically significant heterogeneity. Subgroup analyses were conducted assessing the impact of case type, geographic region, cancer type, TNM stage, age, tumor size, chemotherapy drugs, and time of RFA and follow-up.

## Results

### Results of the search

**Figure 1:**
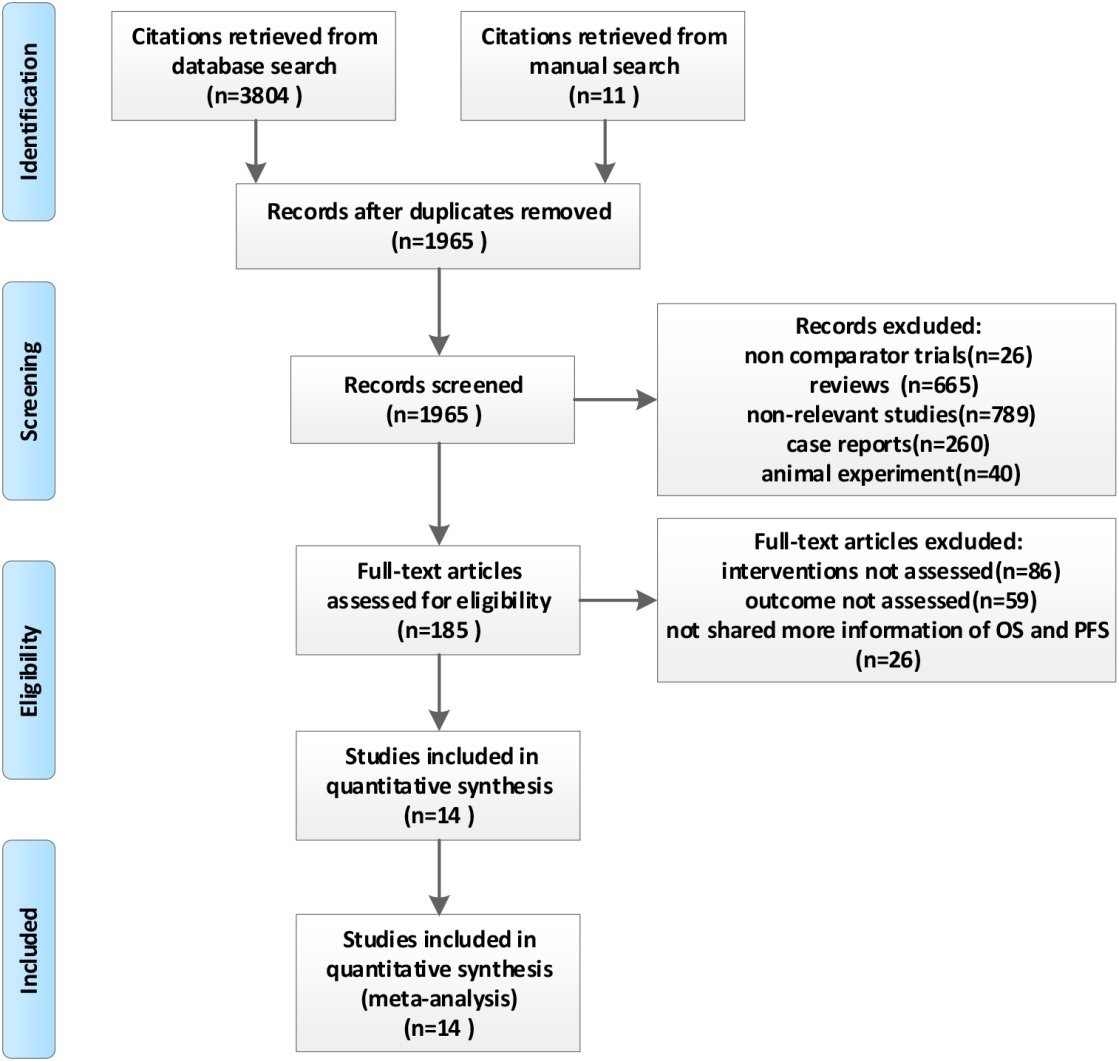
Flow diagram of study selection in the meta-analysis.

**Figure 1** is a flowchart of the literature retrieval. Our literature identified 3,804 records from the database search results, and 11 additional articles were identified from manual searches. After removing the duplicate publications, 1,965 unique references were screened for eligibility by titles and abstracts. The remaining 185 publications were retrieved as full text or abstracts for detailed evaluation. Another 171 articles were excluded for the following reasons: interventions not assessed (n = 86), outcomes not assessed (n = 59), and not sharing more information about HR, OS, and PFS (n = 26). Finally, 14 trials representing 1,387 patients were included in this meta-analysis (11, 12, 23–34).

### Study description

The characteristics of the studies in the evidence synthesis were summarized in **Tables 1**, **2**. All of the studies were non-randomized comparisons. Nine studies were published from 2014 to 2022, and five were published before 2014. Most studies (11/14, 78.6%) were based in China. The sample size ranged from 29 to 256 patients. For NSCLC, the main lung cancer type, nine (n = 768) trials were only focused on NSCLC (23, 26–32, 34), and only one (n = 100) included patients with pulmonary metastases (11). Most patients in the trials were diagnosed with stage III or IV. Eleven studies were included in the comparison of RFA plus chemotherapy vs. chemotherapy alone (n = 1,010) (12, 23–31, 34). Four trials were included in the comparisons of RFA plus chemotherapy vs. RFA alone (n = 320) (11, 26, 32, 33). Only one study compared RFA with chemotherapy (n = 95) (26). One study included those three comparisons with RFA plus chemotherapy (n = 43), chemotherapy (n = 61), and RFA (n = 34) (26). Three studies (11, 31, 33) supplied HR in the articles; others were calculated by OS. Only one trial supplied the PFS curve (12).

**Table 1:** Characteristics of the trials.

Reference	Inclusion period	RFA schedule	Chemotherapy schedule	Sample size	Follow-up (months)
RFA plus chemotherapy vs. chemotherapy					
Xu et al., 2022 (12)	2017-2019	CT-guided RFA, given before chemotherapy; 60–100 W, 10.5 min, 85.0°C ± 15.0°C	Four cycles: paclitaxel 135 mg/m^2^ days 1 and 8; cisplatin 60 mg/m^2^ day 1, every 21 days	256	Median: 16.5 Range: 2–24
Yu, 2020 (23)	2013-2014	CT-guided RFA; 15 KJ, 10 min, approximately 85.0°C	Gemcitabine 1,250 mg/m^2^ days 1 and 8; cisplatin 60 mg/m^2^ days 2–4	80	Range: 6–36^*^
Sun et al., 2019 (24)	2012-2016	Color Doppler ultrasonography-guided RFA	Etoposide 100 mg/m^2^ days 1–3; cisplatin 75 mg/m^2^ day 1 or carboplatin ACU 5–6 day 1, every 21 days	54	Range: 1–18.5^*^
Chen et al., 2018 (25)	2014-2017	CT-guided RFA, given before chemotherapy; 10–15 min, 90°C	Six cycles: gemcitabine 1,000 mg/m^2^ days 1 and 8; cisplatin 30 mg/m^2^ days 1–3, every 21 days	80	Median: 21 Range: 6–32
Du et al., 2017 (34)	2012-2015	CT-guided RFA; 25.32 ± 8.16 min, 90.1°C ± 1.71°C	Vinorelbine-cisplatin or paclitaxel-cisplatin/carboplatin or gemcitabine-cisplatin/carboplatin or docetaxel-cisplatin/carboplatin or pemetrexed-cisplatin/carboplatin	133	Median: 31 Range: 1–43^*^
Yang et al., 2016 (26)	2011-2014	2–3 cycles: CT-guided RFA, after artery chemoembolization 3–7 days, 15 KJ; every 30 days	Two to three cycles: artery chemoembolization, gemcitabine (1.2–2.0 g) and cisplatin (60–80 mg) day 1, every 21–28 days	104	Range: 1–36^*^
Zhou et al., 2015 (27)	2011-2013	1 cycle: CT-guided RFA, in the middle of chemotherapy	Two to six cycles: docetaxel-cisplatin or gemcitabine-carboplatin, every 21 days	122	Median 22.8 Range: 6–36
Zhu et al., 2014 (28)	2010-2012	CT-guided RFA, given before chemotherapy 7 days; 15 min, 90°C	Four cycles: gemcitabine 1,000 mg/m^2^ days 1 and 8; cisplatin 30 mg/m^2^ days 1–3, every 21 days	55	Range: 1–24^*^
Pu et al., 2013 (29)	2009-2012	CT-guided RFA, given before chemotherapy 7 days; 10–15 min, 90°C	Gemcitabine 1,000 mg/m^2^ day 1 and 8, cisplatin 30 mg/m^2^ days 1–3, every 21 days	32	Range: 6–24^*^
Lee et al., 2012 (30)	2000-2004	CT-guided RFA, given before chemotherapy; 60–70 W, 6–12 min	Three to nine cycles: gemcitabine-cisplatin or Taxol-carboplatin	30	Range: 6–48^*^
Wang et al., 2005 (31)	1999-2004	CT-guided RFA, given before chemotherapy 7–15 days	Three to six cycles: gemcitabine 1,250 mg/m^2^ days 1 and 8, cisplatin 75 mg/m^2^ day 1, every 21 days	64	Range: 3–36^*^
RFA plus chemotherapy vs. RFA					
Wang et al., 2020 (32)	2013-2015	2–3 cycles; CT-guided RFA day 1, every 30 days	Two to three cycles: artery chemoembolization, gemcitabine (1.2–2.0 g) and cisplatin (60–80 mg) day 1, every 21–28 days	114	Range: 1–36^*^
Yang et al., 2016 (26)	2011-2014	1–3 cycles: CT-guided RFA, 15 KJ, every 30 days	Two to three cycles: artery chemoembolization, before RFA 3–7 days, gemcitabine (1.2–2.0 g) and cisplatin (60–80 mg) day 1, every 21–28 days	77	Range: 1–36^*^
Chua et al., 2010 (11)	2000-2009	CT-guided RFA; 35–150 W, 90°C, 15–37 min	5-fluorouracil–based chemotherapy or capecitabine	100	Median: 23 Range: 1–96
Sun et al., 2010 (33)	2007-2009	CT-guided RFA; 5–15 min	Artery chemoembolization, before or after RFA 7 days, cisplatin 100 mg, epirubicin 40 mg, vincristine 2 mg	29	Range: 1–20^*^
RFA vs. chemotherapy					
Yang et al., 2016 (26)	2011-2014	1–3 cycles: CT-guided RFA, 15 KJ, every 30 days	Two to three cycles: artery chemoembolization, Gemcitabine (1.2–2.0 g) and cisplatin (60–80 mg) day1, every 21–28 days	95	Range: 1–36^*^

RFA, radiofrequency ablation; CT, computed tomography.

*Unpublished, retrieved from the survival curve.

**Table 2:** Characteristics of patients and outcome summary of studies.

Reference	Type	Phase	Test group							Control group					
			N	Age	Male %	Size cm	Median OS, mo	Overall mortality		N	Age	Male %	Size cm	Median OS, mo	Overall mortality
RFA plus chemotherapy vs. chemotherapy															
Xu et al., 2022 (12)	NSCLC, SCLC, brain metastases	I - IV	128	48.69 ± 6.74	53.90	2.54 ± 0.36	17.5	12 mo: 92.82%*		128	48.69 ± 6.74	52.30	2.54 ± 0.36	13.4	12 mo: 67.13%*
Yu, 2020 (23)	NSCLC	III, IV	40	60.49 ± 4.28	67.50	31.93 ± 4.20^a^	None	12 mo: 92.60%* 24 mo: 92.60%* 36 mo: 87.50%*		40	61.08 ± 5.01	72.50	32.07 ± 3.97^a^	36.0*	12 mo: 85.01%* 24 mo: 65.10%* 36 mo: 50.01%*
Sun et al., 2019 (24)	SCLC, liver metastases	IV	24	60^c^	54.17	≤3.0: 60% <5.0: 40%	10.7	12 mo: 33.27%*		30	64^c^	60.00	≤3.0: 75% <5.0: 25%	6.9	12 mo: 3.45%
Chen et al., 2018 (25)	NSCLC, liver and bone metastases	III, IV	40	60.5 ± 11.9	65.00	4.34 ± 1.35	14.5	12 mo: 74.21%* 24 mo: 10.14%*		40	59.7 ± 11.2	57.50	4.34 ± 1.51	8.4	12 mo: 27.54%* 24 mo: 3.34%*
Du et al., 2017 (34)	NSCLC	III, IV	77	61.8	61.04	4.2	22.1	12 mo: 70.74% 24 mo: 39.31%		56	62.2	71.43	3.7	18.1	12 mo: 54.54% 24 mo: 19.49%
Yang et al., 2016 (26)	NSCLC	III, IV	43	67.2 ± 1.9	67.44	4.8 ± 0.3^b^	21	12 mo: 90.7% 24 mo: 58.1% 36 mo: 20.9%		61	68.2 ± 1.8	65.57	5.1 ± 0.4^b^	14	12 mo: 57.4% 24 mo: 24.6% 36 mo: 11.5%
Zhou et al., 2015 (27)	NSCLC	III, IV	48	60.31 ± 8.56	64.58	4.12 ± 1.47	13.9	12 mo: 61.29%*		74	63.15 ± 9.49	75.68	4.89 ± 1.63	8.12	12 mo: 22.66%*
Zhu et al., 2014 (28)	NSCLC	III, IV	21	70 ± 12	76.19	5.9 ± 2.7	None	12 mo: 90.55%* 24 mo: 85.81%*		34	69 ± 14	67.65	6.0 ± 2.9	None	12 mo: 70.65%* 24 mo: 58.73%*
Pu et al., 2013 (29)	NSCLC	III, IV	16	61(48-76)^d^	75.00	3.1-10.4	18	12 mo: 81.01%* 24 mo: 5.33%*		16	61(45-78)^d^	87.50	3.2-9.8	15	12 mo: 56.19%* 24 mo: 1.14%*
Lee et al., 2012 (30)	NSCLC	III, IV	12	69.4 ± 6.4	83.30	4.6 ± 1.6	42	12 mo: 100% 24 mo: 83.3% 36 mo: 0%		18	67.6 ± 5.2	77.80	5.2 ± 0.3	29	12 mo: 77.8% 24 mo: 63.3% 36 mo: 0%
Wang et al., 2005 (31)	NSCLC	III, IV	34	64.4(45-81)^d^	82.35	5.6-12.4	17.4	12 mo: 76.5% 24 mo: 44.1% 36 mo: 17.6%		30	62.7(42-70)^d^	83.33	None	9.2	12 mo: 43.3% 24 mo: 13.3% 36 mo: 0%
RFA plus chemotherapy vs. RFA															
Wang et al., 2020 (32)	NSCLC	III, IV	61	59.45 ± 6.23	63.93	4.82 ± 0.44^b^	28	12 mo: 90.16% 24 mo: 57.38% 36 mo: 32.79%		53	60.25 ± 5.88	62.26	4.93 ± 0.56^b^	15.0*	12 mo: 58.49% 24 mo: 32.08% 36 mo: 11.32%
Yang et al., 2016 (26)	NSCLC	III, IV	43	67.2 ± 1.9	67.44	4.8 ± 0.3^b^	21	12 mo: 90.7% 24 mo: 58.1% 36 mo: 20.9%		34	67.7 ± 2.0	61.76	4.6 ± 0.6^b^	15.0	12 mo: 58.8% 24 mo: 32.4% 36 mo: 11.8%
Chua et al., 2010 (11)	Colorectal pulmonary metastases	I-IV	59	65 ± 11^e^	None	≤5.0	None	12 mo: 96.36%* 24 mo: 83.51%* 36 mo: 62.56%*		41	65 ± 11^e^	None	≤5.0	20.8*	12 mo: 74.18%* 24 mo: 43.06%* 36 mo: 25.18%*
Sun et al., 2010 (33)	Unclassified	III, IV	14	54(40-71)^d^	71.43	None	None	12 mo: 85%		15	54(41-76)^d^	66.67	None	18.2*	12 mo: 76%
RFA vs. chemotherapy															
Yang et al., 2016 (26)	NSCLC	III, IV	34	67.7 ± 2.0	61.76	4.6 ± 0.6^b^	15	12 mo: 58.8% 24 mo: 32.4% 36 mo: 11.8%		61	68.2 ± 1.8	65.57	5.1 ± 0.4^b^	14	12 mo: 57.4% 24 mo: 24.6% 36 mo: 11.5%

RFA, radiofrequency ablation; OS, overall survival; NSCLC, non-small cell lung cancer; SCLC, small cell lung cancer; N, number; mo, month.

*Estimated as the overall survival curve; ^a^, area of the tumors, cm^2^; ^b^, the maximum size of tumors; ^c^, median age; ^d^, mean age and range; ^e^, mean age and standard deviation for test and control group.

### Risk of bias assessment

**Supplementary Figures S1**, **S2** provide the Cochrane risk of bias. The NOS results are listed in **Supplementary Table S2**. The majority of the included studies were felt to have a low risk of bias. The adequacy of follow-up was often not described in the included studies, which raises the question of bias.

### Survival analysis of RFA plus chemotherapy vs. chemotherapy

**Figure 2:**
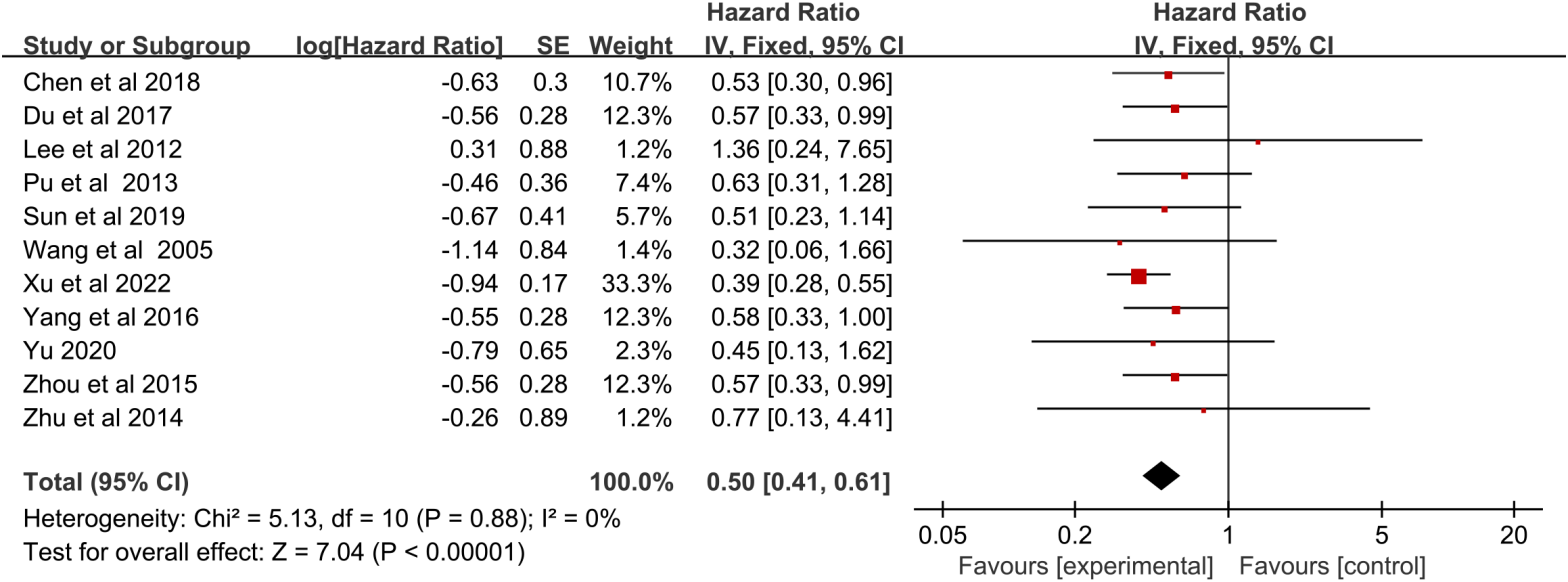
Overall survival for radiofrequency ablation plus chemotherapy compared with chemotherapy alone.

**Figure 3:**
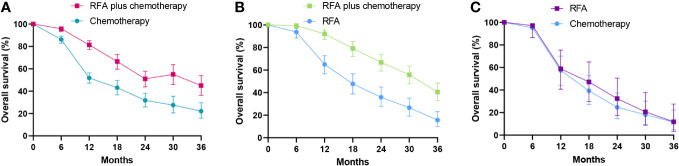
Comparison of overall survival curves for radiofrequency ablation and/or chemotherapy. **(A)** Overall survival curves of radiofrequency ablation plus chemotherapy compared with chemotherapy alone. **(B)** Overall survival curves of radiofrequency ablation plus chemotherapy compared with radiofrequency ablation alone. **(C)** Overall survival curves of radiofrequency ablation compared with chemotherapy. RFA, radiofrequency ablation.

A significant benefit of OS was observed in favor of RFA plus chemotherapy vs. chemotherapy (HR 0.50, 95% CI 0.41–0.61; p < 0.00001) (**Figure 2**). This benefit corresponded to a 50% reduction in the risk of dying and an absolute benefit of 29.6% (95% CI 23.7–35.5), 19.2% (95% CI 10.1–28.2), and 22.9% (95% CI 12.0–33.7) at 12 months, 24 months, and 36 months, respectively (**Figure 3A**). Heterogeneity between trials was not significant (χ^2^ = 5.13, p = 0.88, I^2^ = 0%). We further performed the subgroup analysis, as shown in **Table 3**, and the primary result was independent of case type, cancer type, chemotherapy drugs, and tumor size. However, no statistically significant correlation was found in the Korean population (p = 0.72), TNM stage IV (p = 0.10), age ≥65 years (p = 0.08), RFA in or after the chemotherapy (p = 0.05), and follow-up periods longer than 36 months (p = 0.07) (**Table 3**).

**Table 3:** Subgroup analysis of RFA plus chemotherapy vs. chemotherapy.

Subgroup factors	No. of studies	No. of patients	Effect model	HR(95% CI)	p	Heterogeneity	
						I^2^ (%)	p
**Total**	11	1,010	Fixed	0.50 (0.41-0.61)	<0.00001	0	0.88
Case type							
RCTs	8	192	fixed	0.56 (0.36-0.85)	0.007	0	0.89
Cohort studies	3	818	fixed	0.49 (0.39-0.61)	<0.00001	0	0.71
Geographic region							
China	10	980	Fixed	0.50 (0.41-0.60)	<0.00001	0	0.57
South Korea	1	30	–	1.36 (0.24-7.65)	0.72	–	–
Cancer type							
NSCLC	8	620	Fixed	0.57 (0.43-0.77)	0.0002	0	0.95
Multiple	3	390	Fixed	0.43 (0.33-0.57)	<0.00001	0	0.6
TNM stage							
I-IV	1	256	–	0.39 (0.28-0.55)	<0.00001	–	–
III IV	9	700	Fixed	0.57 (0.45-0.73)	<0.0001	0	0.99
IV	1	54	–	0.51 (0.23-1.14)	0.1	–	–
Age							
≤60	1	189	–	0.39 (0.28-0.55)	<0.00001	–	–
60-65	7	565	Fixed	0.55 (0.42-0.72)	<0.0001	0	0.99
≥65	3	256	Fixed	0.64 (0.39-1.05)	0.08	0	0.63
Tumor size							
≤3.0 cm	1	256	–	0.39 (0.28-0.55)	<0.00001	–	–
≤5.0 cm	4	389	Fixed	0.55 (0.41-0.75)	0.0001	0	0.99
>5.0 cm	6	365	Fixed	0.61 (0.41-0.91)	0.01	0	0.82
Chemotherapy drugs							
GP	6	415	Fixed	0.56 (0.40-0.77)	0.0004	0	0.98
TP	1	256	–	0.39 (0.28-0.55)	<0.00001	–	–
Other	4	339	Fixed	0.58 (0.41-0.82)	0.002	0	0.79
Time of RFA							
Before chemotherapy	6	517	Fixed	0.46 (0.35-0.60)	<0.00001	0	0.55
In chemotherapy	1	122	–	0.57 (0.33-0.99)	0.05	–	–
After chemotherapy	1	104	–	0.58 (0.33-1.00)	0.05	–	–
Follow-up							
≤24 months	4	397	Fixed	0.44 (0.33-0.58)	<0.00001	0	0.57
24> Time ≤36 months	5	450	Fixed	0.54 (0.40-0.74)	0.0001	0	0.97
>36 months	2	163	Fixed	0.62 (0.37-1.04)	0.07	0	0.35

RFA, radiofrequency ablation; NSCLC, non-small cell lung cancer; GP, gemcitabine and cisplatin; TP, paclitaxel and cisplatin.

### Survival analysis of RFA plus chemotherapy vs. RFA

**Figure 4:**
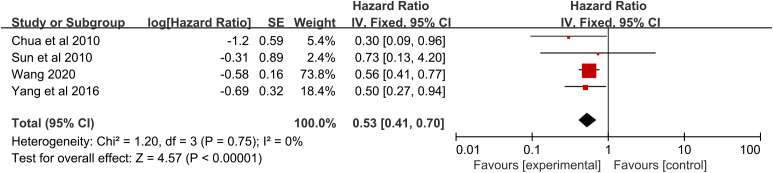
Overall survival for radiofrequency ablation plus chemotherapy compared with radiofrequency ablation alone.

A significant benefit of OS was also observed in favor of RFA plus chemotherapy vs. RFA (HR 0.53, 95% CI 0.41–0.70; p < 0.00001) (**Figure 4**). This benefit corresponded to a 46% reduction in the risk of dying and an absolute benefit of 27.1% (95% CI 18.3–35.8), 31.0% (95% CI 19.9–41.9), and 24.9% (95% CI 15.0–34.7) at 12 months, 24 months, and 36 months, respectively (**Figure 3B**). Heterogeneity between trials was not significant (χ^2^ = 1.20, p = 0.75, I^2^ = 0%). As implied by the subgroup analysis, RFA combined with chemotherapy still significantly improved OS in various subgroups of geographic region and TNM stage (**Table 4**). No statistically significant correlation was detected in RCTs (p = 0.73), size larger than 5.0 cm (p = 0.73), age ≤60 years (p = 0.73), follow-up ≤24 months (p = 0.73), and RFA in the chemotherapy (p = 0.06) (**Table 4**).

**Table 4:** Subgroup analysis of RFA plus chemotherapy vs. RFA.

Subgroup factors	No. of studies	No. of patients	Effect model	HR(95% CI)	p	Heterogeneity		
						I^2^ (%)	p	
**Total**	4	320	Fixed	0.53 (0.41-0.70)	<0.00001	0	0.75
Case type								
RCTs	1	29	–	0.73 (0.13-4.20)	0.73	–	–
Cohort studies	3	291	Fixed	0.53 (0.40-0.70)	<0.00001	0	0.59
Geographic region								
China	3	220	fixed	0.50 (0.41-0.60)	<0.00001	0	0.57
Australia	1	100	–	0.30 (0.09-0.96)	0.04	–	–
Cancer type								
NSCLC	2	191	Fixed	0.55 (0.41-0.73)	<0.0001	0	0.76
Pulmonary metastases	1	100	–	0.30 (0.09-0.96)	0.04	–	–
Unclassified	1	29	–	0.73 (0.13-4.20)	0.73	–	–
TNM stage								
I-IV	3	220	Fixed	0.30 (0.09-0.96)	0.04	–	–
III IV	1	100	–	0.55 (0.42-0.73)	<0.0001	0	0.91
Age								
≤60	1	29	–	0.73 (0.13-4.20)	0.73	–	–
60-65	1	114	–	0.56 (0.41-0.77)	0.0003	–	–
≥65	2	177	fixed	0.45 (0.26-0.78)	0.004	0	0.45
Tumor size								
≤5.0 cm	3	291	Fixed	0.53 (0.40-0.70)	<0.00001	0	0.59
>5.0 cm	1	29	–	0.73 (0.13-4.20)	0.73	–	–
Chemotherapy drugs								
GP	2	191	Fixed	0.55 (0.41-0.73)	<0.0001	0	0.76
Other	2	129	Fixed	0.40 (0.15-1.04)	0.06	0	0.4
Time of RFA								
In chemotherapy	2	129	Fixed	0.40 (0.15-1.04)	0.06	0	0.4
After chemotherapy	1	77	–	0.50 (0.27-0.94)	0.03	–	–
Follow-up								
≤24 months	1	29	–	0.73 (0.13-4.20)	0.73	–	–
24> Time ≤36 months	2	191	Fixed	0.55 (0.41-0.73)	<0.0001	0	0.76
>36 months	1	100	–	0.30 (0.09-0.96)	0.04	–	–

RFA, radiofrequency ablation; NSCLC, non-small cell lung cancer; GP, gemcitabine and cisplatin.

### Survival analysis of RFA vs. chemotherapy

**Figure 5:**
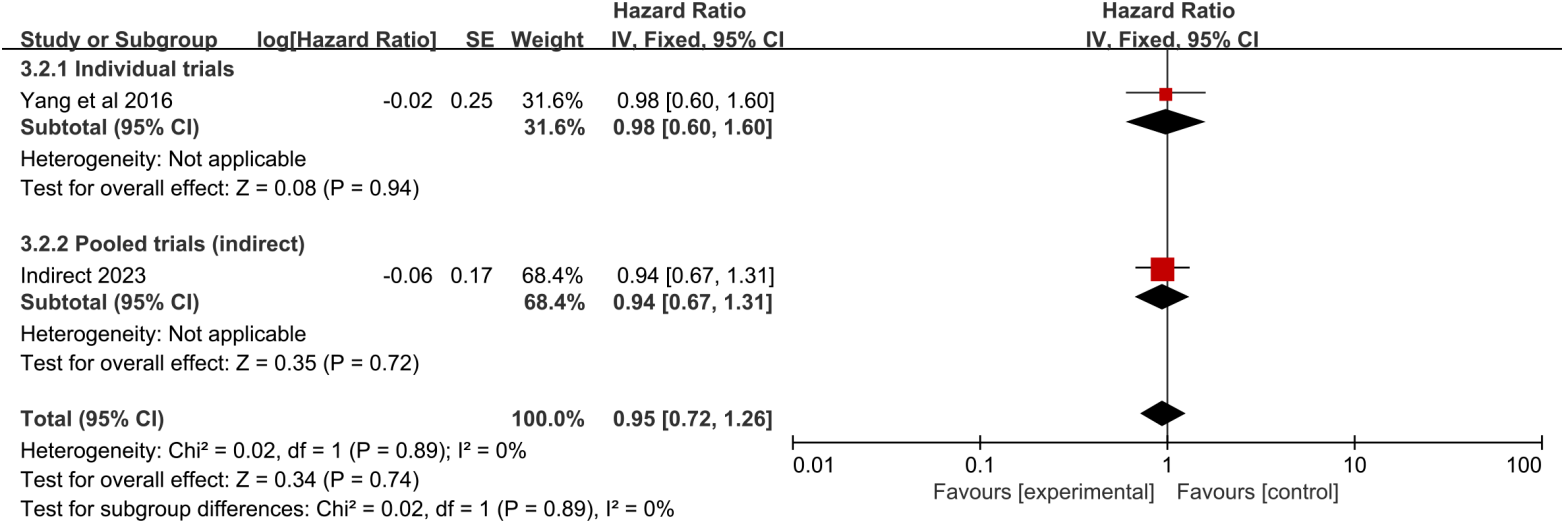
Overall survival for radiofrequency ablation compared with chemotherapy.

The pooled analysis showed that, compared with chemotherapy alone, RFA did not significantly increase OS. There is only one trial evaluating RFA vs. chemotherapy. No significant difference in the survival rate was detected in this trial (HR = 0.98, 95% CI 0.60–1.60; p = 0.94) (**Figure 5**), with an absolute benefit of 1.4% (95% CI -19.2 to 22.1), 7.8% (95% CI -11.3 to 26.8), and 0.3% (95% CI -13.2 to 13.8) at 12 months, 24 months, and 36 months, respectively (**Figure 3C**). From the indirect comparison, the HR was 0.94 (95% CI 0.67–1.31; p = 0.72). Combining the indirect and direct comparisons yielded an overall HR of 0.95 (95% CI 0.72–1.26; p = 0.74) (**Figure 5**). The heterogeneity was not significant (χ^2^ = 0.02, p = 0.89, I^2^ = 0%).

### Published bias analysis

Assessment of publication bias was performed using funnel plots (**Supplementary Figure S3**) and the Egger regression model. The Egger test showed that no publication bias was present for RFA plus chemotherapy vs. chemotherapy (p = 0.0986 > 0.05).

## Discussion

This meta-analysis demonstrates a statistical survival advantage for patients treated with RFA plus chemotherapy compared with those treated with chemotherapy alone or RFA alone, while RFA seems not to significantly improve OS compared with chemotherapy. Our findings provide strong evidence that RFA plus chemotherapy can improve survival in the disease.

To our knowledge, this paper represents the most comprehensive and up-to-date review of the treatment comparisons (RFA plus chemotherapy vs. chemotherapy, RFA plus chemotherapy vs. RFA, RFA vs. chemotherapy) for lung cancer and pulmonary metastases. Few previous meta-analyses have used HR to compare the OS of RFA and/or chemotherapy. Liu et al. (15) found that RFA combination with chemotherapy increased survival rate (response rate (RR) = 1.49, 95% CI 1.35–1.65) and reduced postoperative recrudescence (RR = 0.51, 95% CI 0.32–0.82) compared with chemotherapy for advanced NSCLC. In another meta-analysis (14), the authors also found that RFA plus chemotherapy improved the OS rate compared to chemotherapy alone for advanced NSCLC (1 year, RR = 2.01, 95% CI 1.41–2.86; 2 years, RR = 2.48, 95% CI 1.51–4.07; 3 years, RR = 2.29, 95% CI 1.24–4.22). Peter et al. (16) conducted a meta-analysis of the survival outcomes among lung tumors in nonsurgical patients treated with RFA plus post-ablation chemotherapy vs. RFA. They found that RFA plus post-ablation chemotherapy of lung tumors yielded improved outcomes in terms of local tumor progression, OS, and disease-free survival compared with RFA alone (16). A key limitation of those studies was that they used adjusted and unadjusted odds ratios that do not take into account time-to-over outcome measures.

Since most patients with lung cancer have advanced disease (stage III or IV), they miss the optimal therapeutic window for curative resection. For patients with inoperable cancer, chemotherapy is the mainstay of management. It is a systemic treatment because the chemicals or drugs travel throughout the body and kill cancer cells. The main international guidelines recommend platinum-based chemotherapy as the standard of care for first-line therapy of advanced lung cancer, while those compounds indiscriminately attack all rapidly dividing cells, leading to severe side effects and inducing drug resistance (35). Furthermore, residual tumor cells remain present within resolving lesions after chemotherapy. The posttreatment prognosis of these patients remains poor. The meta-analysis of SCLC suggests that the 6-month survival rate was 75.3% and 72.7% and the 1-year survival rate was 36.2% and 35.0% for cisplatin and carboplatin, respectively (36). Another meta-analysis of NSCLC, which included 38 randomized trials, showed that the 1-year survival rate for the platinum-containing regimens was 34% (37). Thus, more effective and less invasive strategies for advanced lung cancer remain a widespread necessity. As a precise localized and minimally invasive technique, RFA has good safety and effectiveness that can improve the clinical treatment effect and prolong the survival time of patients. It has been widely used in the clinical treatment of lung cancer (38). RFA is also an alternative to surgery for local treatment to eradicate the tumors and has been officially approved as a treatment for NSCLC. The principle of RFA uses high-frequency electromagnetic waves to make intracellular polar molecules agitate and friction to generate heat, leading to protein degeneration and the killing of tumor cells. RFA has an obvious advantage in lung cancer treatment, for a high amount of air in the lung can speed up the accumulation of heat, which causes a rapid temperature increase.

Only one study that met the inclusion criteria for this meta-analysis has directly compared RFA with chemotherapy for NSCLC (26). It seemed that RFA was not significantly beneficial compared with chemotherapy for OS (HR 0.98). Additionally, the absolute difference between 12 months and 36 months was very small in the trial. To help with future trial design and individual patient treatment decisions, we intended to measure the relative survival benefits when these two therapy modalities were directly compared. Weak evidence (5%) in favor of chemotherapy was found in the overall indirect comparison of RFA and chemotherapy (HR 0.95). Shi and Xu (39) compared the survival time and quality of life in patients with lung metastasis from a malignant tumor of the digestive tract between RFA via fiber-optic bronchoscopy and conservative chemotherapy. They reported that the 3-year survival rate with RFA (53.3%) was significantly higher than that of chemotherapy (31.1%) (p < 0.05). Lee et al. (30) reported that the 3-year lung survival rate of NSCLC patients with RFA was 33.3% and chemotherapy alone was 32.4%. The disadvantage may be caused by the fact that RFA effectively kills tumor cells directly. However, this indirect comparison might have been prone to selection bias, and more direct comparisons are needed to test it.

RFA plus chemotherapy provided a better OS. Compared with chemotherapy alone, the HR was 0.50. And compared with RFA alone, the HR was 0.53. We speculate that the improvement in OS of RFA plus chemotherapy compared with RFA alone or chemotherapy alone is due to the following reasons: Firstly, RFA can not only effectively kill tumor cells but also release tumor antigens that can provoke a systemic immune response (40). RFA induces massive necrotic cell death through frictional heating and inflammatory effects. Inflammatory infiltrates that include neutrophils, macrophages, dendritic cells, and natural killer cells are found in the transitional zone. B cells and T cells are specific to the ablated tissue. These immune cell subsets have also been observed in distant untreated tumors and the bloodstream in both patients and animals (41). These results suggest an overall immune activation by RFA. The levels of immunoglobulin A, immunoglobulin G, and immunoglobulin M were increased significantly after RFA (42). The immune response provoked by the localized RFA treatment may have a therapeutic effect on distant primary lesions. This may be the reason why tumor markers decreased after RFA. The combined use of RFA and chemotherapy could decrease further than chemotherapy alone (38). Secondly, chemotherapy resistance limits our ability to effectively treat lung cancer. Some lung tumors are intrinsically resistant to chemotherapy, and in virtually all cases, even the initial responders rapidly develop acquired resistance. RFA induced coagulation necrosis and cell death in the centrally located hypoxic tumor, which is typically less responsive to chemotherapy. RFA, which is a type of hyperthermia, inhibits DNA polymerase-mediated damage repair, increases functional multidrug resistance (MDR) proteins, and abrogates drug resistance (43). In the short-term effect study of middle- and late-period NSCLC, the effective rate of chemotherapy was 27.3%, RFA was 64.3%, and RFA combined with chemotherapy was 80.0%. A previous meta-analysis (15) also indicated that RFA combined with chemotherapy improved short-term effect than chemotherapy (RR = 0.93, 95% CI 0.72–1.20). Thirdly, the goal of combining chemotherapy with thermal ablation is often to enhance tumor cell death in the peripheral or transitional zone, which, at sublethal temperatures, is an area recovering from reversible injury. Apoptosis that is triggered by heat-induced cell injury is increased by the cytotoxic injury of chemotherapies. Chemotherapy is more sensitive to oxygen-enriched cells than to hypoxic cells, while RFA is more sensitive to hypoxic cells. RFA can cause “*in situ* thermal injury” to the large tumor mass, which can lead to a “chemotherapy-sensitizing effect” and make the chemotherapy more effective (44). The synergistic effect of the combination of RFA and chemotherapy has been proven (45). Finally, RFA targets tumors that can be seen in imaging but cannot treat subclinical or small lesions. Moreover, insufficient RFA can lead to the expression of tumor stem markers, promote the generation of tumor stem cells, and further lead to residual cancer recurrence. Chemotherapy is a systemic treatment that has a better effect on subclinical lesions, small lesions, and residual cancer. The benefit of combination survival was also proven in the rabbit VX2 lung tumor model (46). The combination has complementary advantages that increase the disease control rate, objective response rate, and survival (13, 47).

Furthermore, we performed subgroup analyses on case type, therapeutic approaches, and patient characteristics and tried to delve into their applicability. For the Chinese population, a statistically significant correlation was found between the combined application of RFA and chemotherapy vs. sole chemotherapy, whereas this difference was not significant in the Korean population (p = 0.72). Simultaneously, Chinese and Australian patients with lung cancer had comparable outcomes when comparing RFA combined with chemotherapy vs. RFA alone. The literature reports the results of RFA treatment for lung cancer in the United States (48) and Europe (49), but unfortunately, we did not find any trials of relevant comparisons. This may be due to a variety of factors, including the sample size, dissimilarities in populations and biological characteristics of tumors, and disparities in local clinical management (50). It indicated that variances in patient populations across different regions could impact the efficacy of the treatment. The combination of RFA with chemotherapy significantly improved survival compared to chemotherapy alone in different tumor sizes, drugs, and types of lung cancer including NSCLC, SCLC, and pulmonary metastases. Hiraki et al. (51) showed in their meta-analysis that tumor type did not impact local control by RFA in the lungs. Maybe the benefit is derived from the combined synergistic effects of both comprehensive treatment strategies and local therapies.

The subgroup analysis also showed the OS benefit for RFA prior to chemotherapy than for RFA during and after chemotherapy in the comparison of RFA plus chemotherapy and chemotherapy. These findings are comparable to a previously published research by Matsui et al. (52) who retrospectively studied 21 patients who underwent metastasectomy for pulmonary metastases from esophageal cancer. They estimated that 1-year, 3-year, and 5-year survival rates for RFA prior to chemotherapy were 93.3%, 63.8%, and 47.9%, respectively, while those for RFA after chemotherapy were 87.5%, 31.3%, and 31.3% respectively. This difference may be due to the fact that RFA effectively reduces the tumor size, alters the microenvironment of the tumor cells and triggers an immune response, and reduces drug resistance, thus helping to improve the efficacy of subsequent chemotherapy.

When conducting subgroup analyses comparing chemotherapy combined with RFA to RFA, we found tumor size >5.0 cm did differ from the overall analysis. The local efficacy of RFA for the treatment of lung cancer depends on tumor size and the type of electrode used (53). Dupuy et al. (48) reported the results of the American College of Surgeons Oncology Group Z4033 Trial that prospectively evaluated RFA for stage IA NSCLC in medically inoperable patients in 2015. The difference in local control between tumors 3 cm or less in size and tumors larger than 3 cm and therapeutic outcomes are better in smaller cancers. Kodama et al. (54) and Herrera et al. (55) reported a better treatment response in tumors smaller than 5 cm. RFA is strictly dependent on anatomical criteria, such as nodule size and location. Therefore, lesions larger than 5 cm should be excluded from RFA (56).

Our study has certain limitations that should be taken into account. Several studies included in the analysis were not RCTs. Only one retrospective study has been designed to compare chemotherapy directly with RFA for patients with NSCLC (26). Therefore, we conducted a cross-study analysis of data from the comparing of RFA plus chemotherapy with chemotherapy alone and RFA plus chemotherapy with RFA alone. The PFS of those comparisons was also low in the literature. The HR was not directly obtained by the included studies; therefore, it might have led to deviations in the calculated HR. In making treatment decisions, we need to consider toxicity or side effects. However, the adverse events were not analyzed in this paper because the definition, measurement, and reporting of adverse events in the trials were not standard-grade toxicities.

## Conclusion

In conclusion, based on the present evidence, RFA plus chemotherapy improves OS compared with RFA alone or chemotherapy alone. Further research is still needed to compare the efficacy and safety of RFA plus chemotherapy and RFA alone or chemotherapy alone.

## Data availability statement

The original contributions presented in the study are included in the article/**Supplementary Material**. Further inquiries can be directed to the corresponding author.

## Author contributions

All authors were involved in the design of the trial, organization of the study, manuscript writing, and approval of final version of the manuscript. ZY, XL, HY, LH and RZ developed the protocol. ZY, XL, HY, BW, DX, LH and YH generated the search strategy, performed literature search and select. ZY, XL, HY, LH and RZ extracted and summarized the data. ZY did the data analyses, and XL and YH checked the data analysis and validity. ZY, XL, HY, BW, DX, YH and BD interpreted the data. ZY and XL were chief.
