# Supplementary material for: Survival after radiofrequency ablation and/or chemotherapy for lung cancer and pulmonary metastases: a systematic review and meta-analysis
Source: Front Immunol. 2023 Oct 6;14:1240149. doi: 10.3389/fimmu.2023.1240149 (PMC10587578; doi:10.3389/fimmu.2023.1240149)
Supplement: Supplementary file 1 [file DataSheet_1.docx]

Supplementary Material

**Table S1.** Searching strategy for each database

**Table S2.** Newcastle-Ottawa Scale for the risk of bias assessment of non-RCT trials

**Figure S1.** The risk of bias summary with RevMan

**Figure S2.** The risk of bias graph with RevMan

**Figure S3.** Funnel plot of OS for RFA plus chemotherapy versus chemotherapy

# Supplementary Tables

**Table S1. Searching strategy of each database**

**Pubmed**

| **#No** | **Searches** |
| --- | --- |
| #1 | Lung Neoplasms[MeSH Terms] |
| #2 | (Lung cancer[Title/Abstract]) OR (Pulmonary Neoplasms[Title/Abstract]) OR (Neoplasms, Lung[Title/Abstract]) OR (Lung Neoplasm[Title/Abstract]) OR (Neoplasm, Lung[Title/Abstract]) OR (Neoplasms, Pulmonary[Title/Abstract]) OR (Neoplasm, Pulmonary[Title/Abstract]) OR (Pulmonary Neoplasm[Title/Abstract]) OR (Lung Cancer[Title/Abstract]) OR (Cancer, Lung[Title/Abstract]) OR (Cancers, Lung[Title/Abstract]) OR (Lung Cancers[Title/Abstract]) OR (Pulmonary Cancer[Title/Abstract]) OR (Cancer, Pulmonary[Title/Abstract]) OR (Cancers, Pulmonary[Title/Abstract]) OR (Pulmonary Cancers[Title/Abstract]) OR (Cancer of the Lung[Title/Abstract]) OR (Cancer of Lung [Title/Abstract]) |
| #3 | MED19 protein, human[MeSH Terms] |
| #4 | MED19 protein, human[Supplementary Concept] |
| #5 | (pulmonary metastases[Title/Abstract]) OR (Lung metastasis[Title/Abstract]) OR (mediator complex subunit 19, human[Title/Abstract]) OR (LCMR1 protein, human[Title/Abstract]) OR (lung cancer metastasis related protein 1, human[Title/Abstract]) |
| #6 | #1 OR #2 OR #3 OR #4 OR #5 |
| #7 | Radiofrequency Ablation[MeSH Terms] |
| #8 | (radiofrequency ablation [Title/Abstract]) OR (Ablation, Radiofrequency[Title/Abstract]) OR (Radio Frequency Ablation[Title/Abstract]) OR (Ablation, Radio Frequency[Title/Abstract]) OR (Radio-Frequency Ablation[Title/Abstract]) OR (Ablation, Radio-Frequency[Title/Abstract]) OR (RFA[Title/Abstract]) |
| #9 | #7 OR #8 |
| #10 | Drug Therapy[MeSH Terms] |
| #11 | (Drug Therapy[Title/Abstract]) OR (Therapy, Drug[Title/Abstract]) OR (Drug Therapies[Title/Abstract]) OR (Therapies, Drug[Title/Abstract]) OR (Chemotherapy[Title/Abstract]) OR (Chemotherapies[Title/Abstract]) OR (Pharmacotherapy[Title/Abstract]) OR (Pharmacotherapies[Title/Abstract]) |
| #12 | #10 OR #11 |
| #13 | ("1900/01/01"[Date - Publication] : "2023/03/31"[Date - Publication]) |
| #14 | #6 AND #9 AND #12 AND #13 |

**Embase**

| **#No** | **Searches** |
| --- | --- |
| #1 | 'lung cancer'/exp |
| #2 | 'Lung cancer':ab,ti OR 'Pulmonary Neoplasms':ab,ti OR 'Neoplasms, Lung':ab,ti OR 'Lung Neoplasm':ab,ti OR 'Neoplasm, Lung':ab,ti OR 'Neoplasms, Pulmonary':ab,ti OR 'Neoplasm, Pulmonary':ab,ti OR 'Pulmonary Neoplasm':ab,ti OR 'Lung Cancer':ab,ti OR 'Cancer, Lung':ab,ti OR 'Cancers, Lung':ab,ti OR 'Lung Cancers':ab,ti OR 'Pulmonary Cancer':ab,ti OR 'Cancer, Pulmonary':ab,ti OR 'Cancers, Pulmonary':ab,ti OR 'Pulmonary Cancers':ab,ti OR 'Cancer of the Lung':ab,ti OR 'Cancer of Lung ':ab,ti |
| #3 | 'lung metastasis'/exp |
| #4 | 'pulmonary metastases':ab,ti OR 'Lung metastasis':ab,ti OR 'mediator complex subunit 19, human':ab,ti OR 'LCMR1 protein, human':ab,ti OR 'lung cancer metastasis related protein 1, human':ab,ti OR 'MED19 protein, human':ab,ti |
| #5 | #1 OR #2 OR #3 OR #4 |
| #6 | 'radiofrequency ablation'/exp |
| #7 | 'radiofrequency ablation ':ab,ti OR 'Ablation, Radiofrequency':ab,ti OR 'Radio Frequency Ablation':ab,ti OR 'Ablation, Radio Frequency':ab,ti OR 'Radio-Frequency Ablation':ab,ti OR 'Ablation, Radio-Frequency':ab,ti OR 'RFA':ab,ti |
| #8 | #6 OR #7 |
| #9 | 'chemotherapy'/exp |
| #10 | 'Therapy, Drug':ab,ti OR 'Drug Therapies':ab,ti OR 'Therapies, Drug':ab,ti OR 'Chemotherapy':ab,ti OR 'Chemotherapies':ab,ti OR 'Pharmacotherapy':ab,ti OR 'Pharmacotherapies':ab,ti |
| #11 | #9 OR #10 |
| #12 | #5 AND #8 AND #11 |
| #13 | [01-01-1900]/sd NOT [31-03-2023]/sd |

**Web of Science**

| **#No** | **Searches** |
| --- | --- |
| #1 | TS=(Lung cancer OR Pulmonary Neoplasms OR Neoplasms, Lung OR Lung Neoplasm OR Neoplasm, Lung OR Neoplasms, Pulmonary OR Neoplasm, Pulmonary OR Pulmonary Neoplasm OR Lung Cancer OR Cancer, Lung OR Cancers, Lung OR Lung Cancers OR Pulmonary Cancer OR Cancer, Pulmonary OR Cancers, Pulmonary OR Pulmonary Cancers OR Cancer of the Lung OR Cancer of Lung ) |
| #2 | TS=(pulmonary metastases OR Lung metastasis OR mediator complex subunit 19, human OR LCMR1 protein, human OR lung cancer metastasis related protein 1, human ) |
| #3 | #1 OR #2 |
| #4 | TS=(radiofrequency ablation OR Ablation, Radiofrequency OR Radio Frequency Ablation OR Ablation, Radio Frequency OR Radio-Frequency Ablation OR Ablation, Radio-Frequency OR RFA ) |
| #5 | TS=(Therapy, Drug OR Drug Therapies OR Therapies, Drug OR Chemotherapy OR Chemotherapies OR Pharmacotherapy OR Pharmacotherapies) |
| #6 | #3 AND #4 AND #5 |
| #7 | DOP=(1900-01-01/2023-03-31) |
| #8 | #6 AND #7 |

**Cochrane library**

| **#No** | **Searches** |
| --- | --- |
| #1 | MeSH descriptor: [Lung Neoplasms] explode all trees |
| #2 | (Lung Neoplasms):ab,ti,kw OR (Pulmonary Neoplasms):ab,ti,kw OR (Neoplasms, Lung):ab,ti,kw OR (Lung Neoplasm):ab,ti,kw OR (Neoplasm, Lung):ab,ti,kw OR (Neoplasms, Pulmonary):ab,ti,kw OR (Neoplasm, Pulmonary):ab,ti,kw OR (Pulmonary Neoplasm):ab,ti,kw OR (Lung Cancer):ab,ti,kw OR (Cancer, Lung):ab,ti,kw OR (Cancers, Lung):ab,ti,kw OR (Lung Cancers):ab,ti,kw OR (Pulmonary Cancer):ab,ti,kw OR (Cancer, Pulmonary):ab,ti,kw OR (Cancers, Pulmonary):ab,ti,kw OR (Pulmonary Cancers):ab,ti,kw OR (Cancer of the Lung):ab,ti,kw OR (Cancer of Lung ):ab,ti,kw |
| #3 | #1 OR #2 |
| #4 | (pulmonary metastases):ab,ti,kw OR (Lung metastasis):ab,ti,kw OR (mediator complex subunit 19, human):ab,ti,kw OR (LCMR1 protein, human):ab,ti,kw OR (lung cancer metastasis related protein 1, human):ab,ti,kw |
| #5 | #3 OR #4 |
| #6 | (radiofrequency ablation ):ab,ti,kw OR (Ablation, Radiofrequency):ab,ti,kw OR (Radio Frequency Ablation):ab,ti,kw OR (Ablation, Radio Frequency):ab,ti,kw OR (Radio-Frequency Ablation):ab,ti,kw OR (Ablation, Radio-Frequency):ab,ti,kw OR (RFA):ab,ti,kw |
| #7 | MeSH descriptor: [radiofrequency ablation ] explode all trees |
| #8 | #6 OR #7 |
| #9 | MeSH descriptor: [Drug Therapy ] explode all trees |
| #10 | (Therapy, Drug):ab,ti,kw OR (Drug Therapies):ab,ti,kw OR (Therapies, Drug):ab,ti,kw OR (Chemotherapy):ab,ti,kw OR (Chemotherapies):ab,ti,kw OR (Pharmacotherapy):ab,ti,kw OR (Pharmacotherapies):ab,ti,kw OR (Drug Therapy):ab,ti,kw |
| #11 | #9 OR #10 |
| #12 | #5 AND #8 AND #11 limit with Cochrane Library publication date from Jan 1900 to Apr 2023 |

**Scopus**

| **#No** | **Searches** |
| --- | --- |
| #1 | INDEXTERMS("Lung Neoplasms") |
| #2 | TITLE-ABS-KEY("Lung cancer") OR TITLE-ABS-KEY("Pulmonary Neoplasms") OR TITLE-ABS-KEY("Neoplasms, Lung") OR TITLE-ABS-KEY("Lung Neoplasm") OR TITLE-ABS-KEY("Neoplasm, Lung") OR TITLE-ABS-KEY("Neoplasms, Pulmonary") OR TITLE-ABS-KEY("Neoplasm, Pulmonary") OR TITLE-ABS-KEY("Pulmonary Neoplasm") OR TITLE-ABS-KEY("Lung Cancer") OR TITLE-ABS-KEY("Cancer, Lung") OR TITLE-ABS-KEY("Cancers, Lung") OR TITLE-ABS-KEY("Lung Cancers") OR TITLE-ABS-KEY("Pulmonary Cancer") OR TITLE-ABS-KEY("Cancer, Pulmonary") OR TITLE-ABS-KEY("Cancers, Pulmonary") OR TITLE-ABS-KEY("Pulmonary Cancers") OR TITLE-ABS-KEY("Cancer of the Lung") OR TITLE-ABS-KEY("Cancer of Lung ") |
| #3 | #1 OR #2 |
| #4 | INDEXTERMS("pulmonary metastases") |
| #5 | TITLE-ABS-KEY("pulmonary metastases") OR TITLE-ABS-KEY("Lung metastasis") OR TITLE-ABS-KEY("mediator complex subunit 19, human") OR TITLE-ABS-KEY("LCMR1 protein, human") OR TITLE-ABS-KEY("lung cancer metastasis related protein 1, human") OR TITLE-ABS-KEY("MED19 protein, human") |
| #6 | #4 OR #5 |
| #7 | #3 OR #6 |
| #8 | INDEXTERMS("radiofrequency ablation") |
| #9 | TITLE-ABS-KEY("radiofrequency ablation ") OR TITLE-ABS-KEY("Ablation, Radiofrequency") OR TITLE-ABS-KEY("Radio Frequency Ablation") OR TITLE-ABS-KEY("Ablation, Radio Frequency") OR TITLE-ABS-KEY("Radio-Frequency Ablation") OR TITLE-ABS-KEY("Ablation, Radio-Frequency") OR TITLE-ABS-KEY("RFA") |
| #10 | #8 OR #9 |
| #11 | INDEXTERMS("Chemotherapy") |
| #12 | TITLE-ABS-KEY("Therapy, Drug") OR TITLE-ABS-KEY("Drug Therapies") OR TITLE-ABS-KEY("Therapies, Drug") OR TITLE-ABS-KEY("Chemotherapy") OR TITLE-ABS-KEY("Chemotherapies") OR TITLE-ABS-KEY("Pharmacotherapy") OR TITLE-ABS-KEY("Pharmacotherapies") |
| #13 | #11 OR #12 |
| #14 | #7 AND #10 AND #13 |

**Ovid**

| **#No** | **Searches** |
| --- | --- |
| #1 | Lung Neoplasms.sh,ix,at. |
| #2 | (Lung Neoplasms OR Lung cancer OR Pulmonary Neoplasms OR Neoplasms, Lung OR Lung Neoplasm OR Neoplasm, Lung OR Neoplasms, Pulmonary OR Neoplasm, Pulmonary OR Pulmonary Neoplasm OR Lung Cancer OR Cancer, Lung OR Cancers, Lung OR Lung Cancers OR Pulmonary Cancer OR Cancer, Pulmonary OR Cancers, Pulmonary OR Pulmonary Cancers OR Cancer of the Lung OR Cancer of Lung).at,ab,kw. |
| #3 | #1 OR #2 |
| #4 | Lung metastasis.sh,ix,at. |
| #5 | (Pulmonary metastases OR Lung metastasis OR mediator complex subunit 19, human OR LCMR1 protein, human OR lung cancer metastasis related protein 1, human OR MED19 protein, human).at,ab,kw. |
| #6 | #4 OR #5 |
| #7 | #3 OR #6 |
| #8 | Radiofrequency ablation.sh,ix,at. |
| #9 | (radiofrequency ablation OR Ablation, Radiofrequency OR Radio Frequency Ablation OR Ablation, Radio Frequency OR Radio-Frequency Ablation OR Ablation, Radio-Frequency OR RFA).at,ab,kw. |
| #10 | #8 OR #9 |
| #11 | Chemotherapy.sh,ix,at. |
| #12 | (Therapy, Drug OR Drug Therapies OR Therapies, Drug OR Chemotherapy OR Chemotherapies OR Pharmacotherapy OR Pharmacotherapies OR Drug Therapy).at,ab,kw. |
| #13 | #11 OR #12 |
| #14 | #7 AND #10 AND #13 |

**ScienceDirect**

| ("Lung Neoplasms" OR "Lung cancer" OR "Lung metastasis" OR "pulmonary metastases") AND ("radiofrequency ablation " OR "RFA") AND( "Chemotherapy" OR "Drug Therapy") |
| --- |

**SinoMed database** (Translated from Chinese)

| **#No** | **Searches** |
| --- | --- |
| #1 | "Lung Neoplasms"[MeSH:extend] |
| #2 | "Lung Neoplasms"[Normal Text:smart] OR "Lung cancer"[Normal Text:smart] OR "non-small cell lung cancer"[Normal Text:smart] OR "NSCLC"[Normal Text:smart] OR "small cell lung cancer"[Normal Text:smart] OR "SCLC"[Normal Text:smart] |
| #3 | "Lung metastasis"[Normal Text:smart] OR "Pulmonary metastases"[Normal Text:smart] OR "Lung cancer metastasis"[Normal Text:smart] OR "Metastasis to lung"[Normal Text:smart] OR "Metastasis to pulmonary"[Normal Text:smart] |
| #4 | (#1) OR (#2) OR (#3) |
| #5 | "Radiofrequency ablation "[MeSH:extend] |
| #6 | "Radiofrequency ablation "[Normal Text:smart] OR "RFA"[Normal Text:smart] |
| #7 | (#5) OR (#6) |
| #8 | "Chemotherapy"[MeSH:extend] |
| #9 | "Chemotherapy"[Normal Text:smart] OR "Drug Therapies"[Normal Text:smart] |
| #10 | (#8) OR (#9) |
| #11 | (#4) AND (#7) AND (#10) |

**China National Knowledge Infrastructure Database (CNKI)** (Translated from Chinese)

| ((SU=('Lung cancer'+'Pulmonary Neoplasms'+'non-small cell lung cancer'+'NSCLC'+'small cell lung cancer'+'SCLC'+'Lung metastasis'+'Pulmonary metastases'+'Lung cancer metastasis'+'Metastasis to lung'+'Metastasis to pulmonary')) OR (TKA=('Lung cancer'+'Pulmonary Neoplasms'+'non-small cell lung cancer'+'NSCLC'+'small cell lung cancer'+'SCLC'+'Lung metastasis'+'Pulmonary metastases'+'Lung cancer metastasis'+'Metastasis to lung'+'Metastasis to pulmonary'))) AND ((SU=('radiofrequency ablation '+'RFA')) OR (TKA=('radiofrequency ablation '+'RFA'))) AND ((SU=('Chemotherapy'+'Drug Therapies')) OR (TKA=('Chemotherapy'+'Drug Therapies'))) |
| --- |

**Wangfang Database** (Translated from Chinese)

| T(Topic=Lung cancer OR Pulmonary Neoplasms OR non-small cell lung cancer OR NSCLC OR small cell lung cancer OR SCLC OR Lung metastasis OR Pulmonary metastases OR Lung cancer metastasis OR Metastasis to lung OR Metastasis to pulmonary) AND (Topic=Radiofrequency ablation OR Radiofrequency ablation technology OR RFA) AND (Topic=Chemotherapy OR Drug Therapies) |
| --- |

**Chongqing VIP Chinese Science and Technology Periodical Database (VIP)**

(Translated from Chinese)

| (M=(Lung cancer OR Pulmonary Neoplasms OR non-small cell lung cancer OR NSCLC OR small cell lung cancer OR SCLC OR Lung metastasis OR Pulmonary metastases OR Lung cancer metastasis OR Metastasis to lung OR Metastasis to pulmonary))+(R=(Lung cancer OR Pulmonary Neoplasms OR non-small cell lung cancer OR NSCLC OR small cell lung cancer OR SCLC OR Lung metastasis OR Pulmonary metastases OR Lung cancer metastasis OR Metastasis to lung OR Metastasis to pulmonary))) AND ((M=(Radiofrequency ablation OR Radiofrequency ablation technology OR RFA))+(R=(Radiofrequency ablation OR Radiofrequency ablation technology OR RFA))) AND ((M=(Chemotherapy OR Drug Therapies))+(R=(Chemotherapy OR Drug Therapies))) |
| --- |

| **Table S2.** Newcastle-Ottawa Scale for the risk of bias assessment of non-RCT trials | | | | | | | | | |
| --- | --- | --- | --- | --- | --- | --- | --- | --- | --- |
| **Study** | **Selection** | | | | **Comparability** | **Outcome** | | | **Overall** |
|  | **Representativeness of exposed cohort** | **Selection of no-exposed** | **Ascertainment of exposure** | **Outcome not present at start** |  | **Assessment of outcome** | **Adequate follow-up length** | **Adequacy of follow-up** |  |
| Xu et al, 2022 (12) | ☆ | ☆ | ☆ | ☆ | ☆☆ | ☆ | - | ☆ | 8 |
| Wang et al, 2020 (32) | ☆ | ☆ | ☆ | ☆ | ☆☆ | ☆ | ☆ | - | 8 |
| Sun et al, 2019 (24) | ☆ | ☆ | ☆ | ☆ | ☆ | ☆ | - | - | 6 |
| Du et al, 2017 (34) | ☆ | ☆ | ☆ | ☆ | ☆ | ☆ | ☆ | ☆ | 8 |
| Yang et al, 2016 (26) | ☆ | ☆ | ☆ | ☆ | ☆☆ | ☆ | - | - | 7 |
| Zhou et al, 2015 (27) | ☆ | ☆ | ☆ | ☆ | ☆☆ | ☆ | ☆ | - | 8 |
| Zhu et al, 2014 (28) | ☆ | ☆ | ☆ | ☆ | ☆☆ | ☆ | - | - | 7 |
| Lee et al, 2012 (30) | ☆ | ☆ | ☆ | ☆ | ☆☆ | ☆ | ☆ | - | 8 |
| Chua et al, 2010 (11) | ☆ | ☆ | ☆ | ☆ | ☆ | ☆ | ☆ | - | 7 |
| Wang et al, 2005 (31) | ☆ | ☆ | ☆ | ☆ | ☆ | ☆ | ☆ | ☆ | 8 |

# Supplementary Figures

**
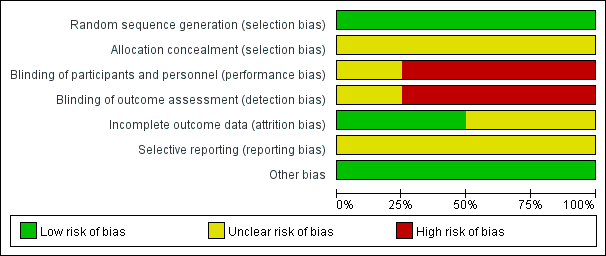
**

**Figure S1.** The risk of bias summary with RevMan

**
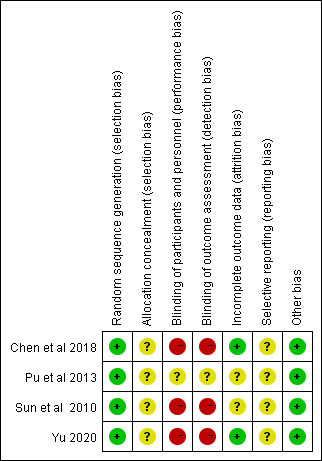
**

**Figure S2.** The risk of bias graph with RevMan


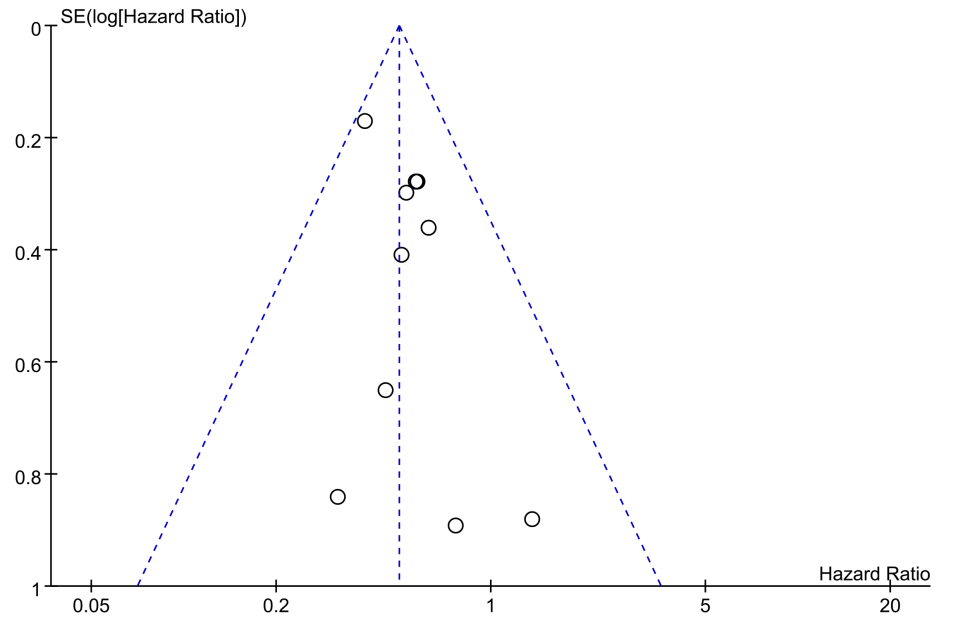


**Figure S3.** Funnel plot of OS for RFA plus chemotherapy versus chemotherapy.
